# Feasibility, Usability, and Preliminary Kinematic Outcomes of a Hand-Tracking Virtual Reality Rehabilitation System Delivered as an Adjunct to Conventional Therapy in Individuals with Stroke: A Single-Arm Pilot Study

**DOI:** 10.3390/healthcare14152373

**Published:** 2026-08-03

**Authors:** Hayati Türe, Eren Kalfa, Osman Topçu, Köksal Sarıhan, Erhan Özdemir, Buket Özdemir Işık

**Affiliations:** 1 Department of Computer Engineering, Trabzon University, Trabzon 61335, Turkey; 2 Department of Digital Game Design, Trabzon University, Trabzon 61335, Turkey; erenkalfa@trabzon.edu.tr; 3 Maçka Ömer Burhanoğlu Physical Therapy and Rehabilitation Hospital, Trabzon 61750, Turkey; topcuosman61@gmail.com (O.T.); koksalsarihan@hotmail.com (K.S.); erhanmagura@gmail.com (E.Ö.); 4 Department of Recreation, Trabzon University, Trabzon 61335, Turkey; ozdemirbuket@trabzon.edu.tr

**Keywords:** virtual reality rehabilitation, upper extremity rehabilitation, stroke, kinematic analysis, preliminary clinical outcomes, neurorehabilitation, task-oriented training

## Abstract

**Highlights:**

**What are the main findings?**

- In this single-arm feasibility study, a consumer-grade hand-tracking virtual reality system delivered as an adjunct to conventional physiotherapy showed good usability and a 71.4% intervention completion rate (10/14 of those who started; 100% per-protocol session adherence among the ten completers); clinical effectiveness was not established and cannot be inferred from this design.
- Continuous in-game kinematic indicators (movement speed and pause duration) emerged as candidate process measures for monitoring engagement and movement behavior across sessions.

**What are the implications of the main finding?**

- Controller-free hand tracking may improve accessibility for patients with grip impairment, but this possibility—and the utility of the in-game kinematic indicators—requires confirmation against gold-standard motion capture and stroke-specific clinical scales in comparative trials.
- Adequately powered randomized controlled trialswith concurrent control of conventional-therapy intensity and stroke-specific upper-extremity scales (e.g., FMA-UE, ARAT) are required before any claim of clinical efficacy.

**Abstract:**

Purpose: Upper extremity motor impairments following stroke substantially limit independence in daily living. Hand-tracking virtual reality (VR) rehabilitation systems may support intensive task-oriented practice while enhancing motivation; however, evidence integrating objective kinematic indicators with usability and patient-reported outcomes for controller-free consumer-grade VR remains limited. The primary aim of this single-arm pilot study was to evaluate the feasibility, safety (tolerability), and usability of a hand-tracking VR rehabilitation system delivered as an adjunct to conventional physiotherapy in individuals with stroke; describing its preliminary in-game kinematic profile and exploring participants’ experiences through open-ended feedback were secondary aims. The study was explicitly not designed or powered to test clinical efficacy. Materials and Methods: Ten individuals with stroke completed a 20-session (8-week) single-arm pilot intervention; all participants concurrently received standard hospital-based physiotherapy (median 3 sessions/week, ∼45 min/session). Early-phase (sessions 1–5) and late-phase (sessions 16–20) within-subject performance were compared using the Wilcoxon signed-rank test with Holm–Bonferroni correction across four pre-specified primary outcomes. Movement smoothness was assessed using the Spectral Arc Length (SPARC), usability was evaluated using the System Usability Scale (SUS), and cybersickness was monitored with the Simulator Sickness Questionnaire (SSQ). Results: Sixteen patients were screened, of whom fourteen started the intervention and ten completed the 8-week per-protocol program (intervention completion rate, 10/14 = 71.4%; per-protocol session adherence among the ten completers, 200/200 = 100%; no SSQ-defined adverse events). Within-subject comparisons showed a +10.6-point increase in success rate (adjusted p=0.020), a +0.10 m/s increase in mean movement speed (adjusted p=0.020), a −341 ms reduction in pause duration (adjusted p=0.022), and a +27.0-point Hodges–Lehmann median-difference increase in SS-QOL (95% CI 14.0–41.0; participant-level median Δ+18.5; adjusted p=0.020). The mean SUS score was 78.5±5.4, indicating “good” usability. Thematic analysis of post-intervention open-ended feedback identified three themes—motivation and engagement, the value of feedback, and design and comfort suggestions—that converged with the high adherence and good usability ratings. Correlations between VR-derived kinematic change and clinical change were non-significant trends (p≥0.12). Conclusions: A controller-free hand-tracking VR system was found to be feasible, well tolerated, and rated as having good usability when delivered as an adjunct to conventional therapy. Because the study used a single-arm design, included only ten participants, and did not control for the confounding effect of concurrent conventional physiotherapy or natural recovery, the observed within-subject changes cannot be causally attributed to the VR intervention and should be interpreted as exploratory feasibility signals. Adequately powered randomized controlled trials with stroke-specific clinical scales (e.g., FMA-UE, ARAT) are required before clinical efficacy can be claimed.

## 1. Introduction

Stroke is a leading cause of disability on a global scale, with more than 12 million new cases annually as of 2019 and a burden affecting the lives of nearly 100 million individuals [1]. Upper-extremity motor deficits following stroke are prevalent and severely limit the independent performance of activities of daily living [2,3]. Current rehabilitation guidelines emphasize that intensive, repetitive, and task-oriented exercises constitute the fundamental approach for supporting motor recovery [4]. In clinical practice, maintaining high-dose therapy is challenging due to resource, time, and motivational constraints. In this context, virtual reality (VR) and gamified interactive exercise have the potential to enhance treatment engagement and make task-based practice more motivating [5]. Systematic reviews and meta-analyses demonstrate that VR-based interventions are associated with significant improvements in upper extremity function [6,7,8].

As emphasized by iterative design and usability studies, VR-based systems must be grounded in user-centered design and acceptability assessments for clinical adoption [9,10]. Studies of patient perceptions and experiences provide critical insights into motivation and sustainable use [11]. Nevertheless, three specific gaps remain in the existing evidence on hand-tracking VR rehabilitation. First, most published systems still rely on hand-held controllers or external optical sensors (e.g., Leap Motion), which limits accessibility for patients with grip impairment [12,13,14]. Second, although SPARC has been shown to be a reliable smoothness metric in stroke populations [15] and consumer-grade headset hand-tracking accuracy is approaching levels acceptable for clinical kinematic measurement [16,17,18], very few clinical reports integrate continuous in-game kinematic data with usability and patient-reported outcomes within the same protocol. Third, feasibility evidence for controller-free consumer-grade hand-tracking VR delivered as an adjunct to standard rehabilitation in heterogeneous post-stroke samples is sparse, leaving uncertainty about adherence, tolerability (cybersickness), and the practical utility of in-game kinematic indicators in routine practice.

The present pilot study addresses these gaps by reporting feasibility and exploratory outcomes from a consumer-grade hand-tracking VR system used in addition to conventional physiotherapy. Consistent with established pilot/feasibility objectives [19,20], the primary aims are to pilot-test the *feasibility*, *safety (tolerability)*, and *usability* of the system rather than its clinical efficacy. Specifically, the study aims to (i) evaluate **feasibility** (recruitment, adherence, and intervention completion); (ii) evaluate **safety and tolerability** (cybersickness assessed with the Simulator Sickness Questionnaire, together with adverse-event and dropout logging) and **usability** (System Usability Scale); (iii) describe, as exploratory secondary outcomes, within-subject changes in pre-specified in-game performance indicators (success rate, mean movement speed, pause duration) and patient-reported quality of life (SS-QOL) over an 8-week, 20-session intervention and characterize the exploratory kinematic profile of movement quality (SPARC, trajectory efficiency, maximum deviation, target overshoot); (iv) describe exploratory associations between in-game kinematic change and standard clinical change; and (v) explore participants’ subjective experiences and acceptability of the system through open-ended feedback analyzed thematically. Given the single-arm design, absence of a control group, small sample size, and concurrent provision of conventional therapy, the study is explicitly framed as a feasibility, safety, and usability investigation; it is not designed or powered to test clinical efficacy, and the exploratory outcomes under aims (iii)–(iv) are reported only to inform the design of a future randomized controlled trial.

## 2. Methods

### 2.1. Clinical Study Sample

This was a prospective, single-arm, single-center pilot feasibility study with a pre–post within-subject comparative design, conducted in a hospital-based clinical rehabilitation setting. The hand-tracking VR rehabilitation system was delivered as an adjunct to ongoing standard physiotherapy; it was not intended to replace conventional rehabilitation. Reporting followed the STROBE checklist [21]; given the digital nature of the intervention, the CONSORT-EHEALTH [22] and TIDieR [23] frameworks were also used to guide the description of the intervention. The study was not designed or powered to evaluate clinical efficacy; the primary aim was to estimate feasibility, usability, and within-subject change parameters that may inform the design of a future randomized controlled trial.

Given the pilot nature of the study, prospective clinical trial registration was not performed; this is acknowledged as a methodological limitation (see Section 5). Protocol development, system finalization, and instrument preparation took place from March 2025 onward. Following approval by the Trabzon University Clinical Research Ethics Committee (Decision No. 2025/08-15, dated 15 August 2025), all participant-facing activities—screening, informed consent, enrollment, the intervention, and data collection—were conducted from 15 August 2025 through 30 April 2026, using consecutive sampling. No participant-facing activity took place before ethics committee approval. Patients meeting the inclusion criteria were invited to participate in order of application.

Individuals with post-stroke upper extremity involvement, aged 18–80 years, with a Brunnstrom upper extremity stage ≥3, and with sufficient cognitive capacity to use the VR system were included. “Sufficient cognitive capacity” was operationally defined as a Mini-Mental State Examination (MMSE) score ≥ 24/30, in accordance with the conventional cutoff for the absence of clinically significant cognitive impairment [24,25]. Brunnstrom staging classifies post-stroke motor recovery through sequential stages, with stage ≥3 representing the level at which voluntary movement begins [26]. Exclusion criteria were severe cognitive impairment (MMSE < 24/30), uncontrolled neurological comorbidities, upper extremity surgery within the previous six months, or severe visual or hearing impairment that would preclude use of the head-mounted display.

Sample size was not formally calculated; n = 10 was selected as a feasibility sample, consistent with current pilot-trial guidance suggesting 9–15 participants per arm as a pragmatic minimum to estimate feasibility parameters and within-subject variability [19,20,27]. Therefore, this sample size is appropriate for feasibility estimation, but by design is insufficient to support inferential conclusions regarding clinical efficacy.

### 2.2. Hand-Tracking VR Rehabilitation System

**Figure 1:**
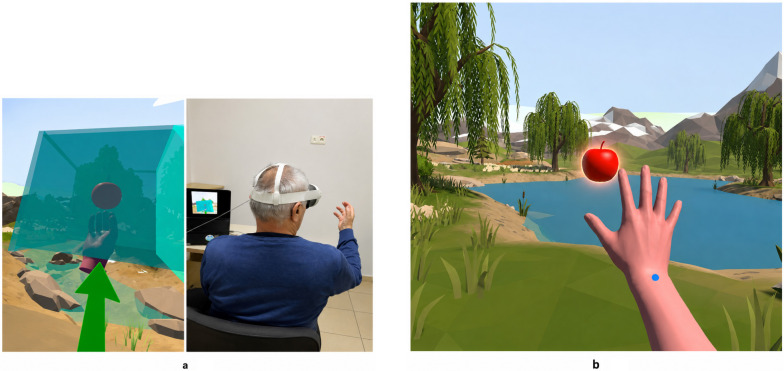
Virtual reality rehabilitation system: (**a**) hand tracking-based therapy environment with Meta Quest 3; (**b**) in-game screenshot showing upper-extremity exercise using a fruit-picking metaphor.

The hand-tracking VR rehabilitation system (henceforth “the VR system”) is built on the Meta Quest 3 (Meta, a product manufactured in Menlo Park, CA 94025, USA) head-mounted display and supports seven gamified therapy modes (Reach, Grip, Carry, Sort, Reflex, Moving Targets, Glass Cube) (Figure 1). The system collects kinematic data at a sampling frequency of 72–90 Hz and automatically computes movement smoothness and efficiency metrics. A web-based therapist panel provides session-level visualization and progress tracking. Throughout this manuscript the single term “the hand-tracking VR rehabilitation system” is used; “hand-tracking-assisted game-based VR” and “VR serious game” have been removed to ensure terminological consistency.

### 2.3. Hand-Tracking Technical Specifications

The Meta Quest 3 head-mounted display (HMD) employs multiple cameras and depth sensors using an inside-out tracking approach. Hand-tracking accuracy and latency values depend on measurement conditions; evaluations on Quest 3 and Quest 2 have reported centimeter-level position errors and latencies of tens of milliseconds [17,18]. Via the XR_EXT_hand_tracking extension conforming to the OpenXR standard, 26 tracking points (XR_HAND_JOINT_COUNT_EXT = 26) are tracked for each hand [28,29]. These points comprise the wrist (1 point), palm (1 point), thumb metacarpal and phalanges (4 points), and for the other four fingers the metacarpal, proximal, middle, and distal phalanges plus fingertip (5×4=20 points).

The dataset recorded at each sampling moment is defined as follows:

(1) $$S_{t}=\left\{p_{t},\;q_{t},\;g_{t},\;t,\;id_{t}\right\}$$

where $p_{t}$ is the three-dimensional hand position vector (m), $q_{t}$ is hand orientation in unit quaternion form, $g_{t}$ is grip state (0: open, 1: closed), *t* is timestamp (ms), and $id_{t}$ is the interacted target object identifier.

### 2.4. System Architecture and Components

**Figure 2:**
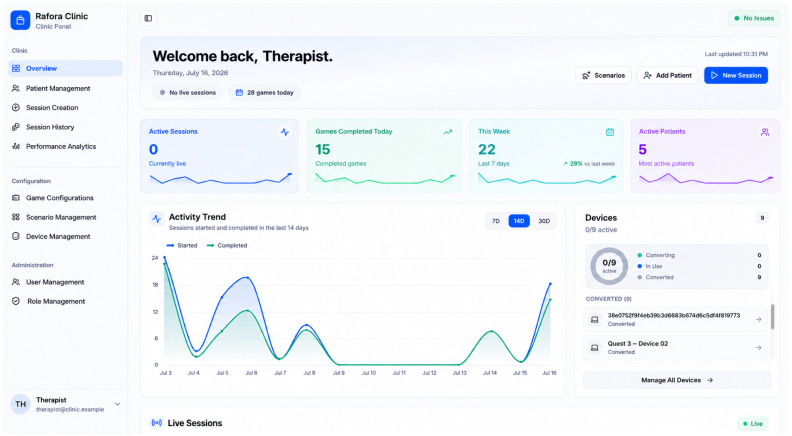
Therapist web panel interface displaying real-time patient performance monitoring, session management controls, and progress visualization dashboard.

**Figure 3:**
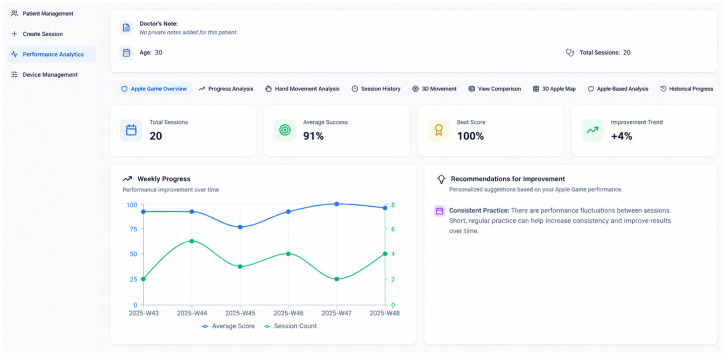
Performance analysis graphs illustrating task metrics including movement speed, trajectory efficiency, and success rate trends across intervention sessions.

The system comprises three main layers: (1) the VR client layer (Unity-based therapy game), (2) the web client layer (therapist panel), and (3) the server and analytics layer (API, real-time communication, and analytics). The VR application tracks hand movements in real time, executes game scenarios, and transmits session telemetry to the server. The therapist panel provides patient/session management, live monitoring, and reporting functions. The server and analytics layer includes components for authentication, patient/session management, data storage, real-time communication, and kinematic metric computation (Figure 2 and Figure 3). Figure 2 shows the general view of the therapist panel, and Figure 3 shows examples of session performance graphs.

### 2.5. Camera Reference System

*In plain terms.* Because each patient sits in a slightly different position relative to the headset, the raw 3D hand-tracking coordinates are not directly comparable across sessions. To make them comparable, the system re-expresses all hand positions in a reference frame anchored to the patient’s own head position and forward-facing direction at the start of each session. This standard rigid-body kinematics operation is described mathematically below for replicability; the clinically relevant point is that all reported kinematic values are in this patient-centered reference frame.

An affine transformation-based reference coordinate system was implemented to eliminate coordinate inconsistencies arising from different patient seating positions. At game initiation, the camera (HMD) position and Y-axis rotation are taken as reference. The use of rotation and homogeneous transformation matrices is a standard method in rigid-body kinematics [30].

Reference rotation matrix:

(2) $$R_{\mathrm{ref}}=\left[\begin{array}{ccc} \cos\theta_{y} & 0 & \sin\theta_{y}\\ 0 & 1 & 0\\ -\sin\theta_{y} & 0 & \cos\theta_{y} \end{array}\right]$$

and homogeneous transformation matrix:

(3) $$T_{\mathrm{ref}}={\left[\begin{array}{cc} R_{\mathrm{ref}} & p_{\mathrm{cam}}\\ 0^{T} & 1 \end{array}\right]}_{4\times 4}$$

with transformation from world coordinates to reference coordinates:

(4) $$p_{\mathrm{ref}}=T_{\mathrm{ref}}^{-1}\;\cdot \;p_{\mathrm{world}}.$$

This transformation preserves only the rotation around the Y-axis, ignoring forward–backward tilt (pitch) and lateral tilt (roll) angles.

### 2.6. Data Flow and Analytics Pipeline

**Figure 4:**
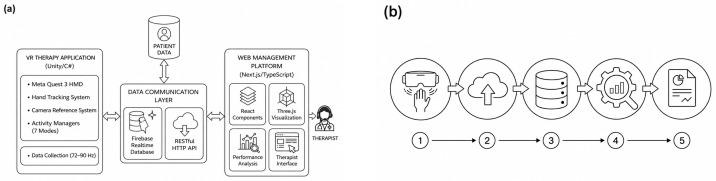
Technical architecture: (**a**) data flow between the VR client, web client, and server layers; (**b**) analytics pipeline from VR device to server, analysis, and report.

Session data are transmitted from the VR device to the server in real time, with high-frequency hand movement data stored in time series format and processed by the analytics service. The analytics pipeline separates the grab and basket (carry) phases for each game task and calculates the speed, efficiency, and smoothness metrics (Figure 4). These metrics are based on kinematic components commonly reported in post-stroke reaching tasks [31].

Analytics pipeline (Figure 4b) steps:

1. Collection and time-stamped recording of raw hand-tracking data;
2. Calibration and preprocessing (noise reduction, missing sample check);
3. Separation of task phases (grab and carry);
4. Calculation of kinematic metrics (speed, efficiency, smoothness);
5. Reporting results and visualization to therapist panel.

### 2.7. Game Modules and Gamification Design

The system is built upon a VR game module called the Functional Reach & Grasp Task (FRGT). This module targets upper extremity rehabilitation using a fruit-harvesting metaphor. The FRGT consists of tasks including reaching, gripping, carrying, sorting, reflex, moving targets, and precise positioning (Table 1). This module supports motor control and coordination at multiple difficulty levels. Parameters such as difficulty level (easy–medium–hard), target distance, task count, and time limit for each mode can be configured by the therapist.

The system structures rehabilitation exercises through gamification principles [32]. Core gamification elements include goal setting, instant feedback, scoring, level/difficulty progression, audiovisual rewards, performance summary, and progress tracking. These elements are designed to support repetition, task orientation, and motivation in alignment with motor learning principles [33]. Gamification mechanics include:

- **Scoring:** Performance score from weighted combination of efficiency, speed, and smoothness metrics.
- **Visual/Auditory Feedback:** Instant feedback for events such as target capture, correct basket, and fast reaction.
- **Progression:** Easy–medium–hard difficulty levels adjusted by target distance, time, and task count.
- **Goal Clarity:** Clear success criteria for each task (touch, grip, carry, correct placement).
- **Progress Tracking:** Inter-session performance graphs and trends displayed in the therapist panel.

This gamification approach increases motivation and task continuity while facilitating data-driven decision-making by therapists.

### 2.8. Therapist Workflow

The therapist panel includes patient selection, game/session planning, live monitoring, and report generation steps. During live monitoring, instant speed, efficiency, and task success rates are displayed; post-session summary reports are generated automatically. Software version and hardware configuration: VR application Unity-based therapy game (v1.0.0; compilation date: 15 January 2025); therapist panel web application (v1.0.0); device Meta Quest 3 (hand tracking 2.3). Sessions were conducted in a quiet room with fixed lighting, in a seated position; a brief calibration routine was performed at the beginning of each session: (i) headset position and user seating were stabilized, (ii) hand-tracking accuracy was verified using the target reference point, and (iii) camera (HMD) position and Y-axis rotation were taken for the reference coordinate system. When calibration failed, it was repeated before session initiation. The system provides access control through role-based authorization; personal data were encrypted for storage, and data shared for research purposes were anonymized.

The study protocol consisted of 2–3 sessions per week under physiotherapist supervision; each session lasted 15–20 min, for a total of 20 sessions over approximately 8 weeks. All participants concurrently received standard hospital-based physiotherapy, scheduled on different days from the VR sessions; no element of the conventional rehabilitation program was withheld or modified for the study.

Pre-specified primary outcomes (rationale).

Four pre-specified primary outcomes were selected on the basis that each captures a distinct and complementary feasibility-relevant construct: *(i)* task **success rate** as a process-level indicator of in-game performance and engagement [31]; *(ii)* **mean movement speed** as a continuous and robust kinematic indicator that consistently differentiates stroke-affected from typical reaching [31,34]; *(iii)* **pause duration** as an indicator of movement segmentation and arrest periods with sensitivity to motor recovery [34,35]; and *(iv)* the **Stroke-Specific Quality of Life Scale (SS-QOL)** as the only patient-reported outcome with established psychometric properties in stroke populations [36]. These four outcomes were specified prior to data analysis and form the basis of the multiple-comparisons correction (Section 2.10).

Secondary (exploratory) outcomes.

Secondary outcomes were SPARC, trajectory efficiency, maximum deviation, and target overshoot [15,34,37]. They are reported descriptively without inferential testing or correction for multiplicity in order to characterize the kinematic profile of change.

Operational definitions.

Success rate was defined as the ratio of successfully completed tasks to total task count [31]. Mean speed was the average velocity magnitude of the hand endpoint over the active task interval. Pause duration was the cumulative time during which the hand endpoint speed remained below an a priori threshold of 0.05 m/s for at least 200 ms; this threshold is consistent with prior reach-to-grasp kinematic literature in stroke [34,35]. SS-QOL is a 49-item self-report scale (12 domains; total score range 49–245); higher scores indicate better quality of life [36].

Usability, tolerability, and adverse events.

Usability was assessed using the 10-item System Usability Scale (SUS), scored on the standard 0–100 range [38]. Cybersickness and intervention tolerability were assessed using the Simulator Sickness Questionnaire (SSQ) [39], administered at three predefined time points (baseline, mid-protocol (session 10), and end of protocol (session 20)) and additionally whenever participants spontaneously reported discomfort. An SSQ total score > 15 or any individual symptom rated as “severe” was pre-specified as a clinically relevant adverse event triggering session interruption. Adverse events, dropouts, and protocol deviations were logged at every session.

Qualitative usability feedback (open-ended responses).

To contextualize and help interpret the quantitative usability and feasibility data, a brief qualitative component was nested within the feasibility study. After completing the SUS at the end of the protocol, the ten completers were each asked two open-ended questions: *(Q1)* “What did you like about the system and what motivated you while using it, and how did it feel to use?” and *(Q2)* “What difficulties did you experience, and what would you change or improve?”. Responses were recorded verbatim; because participants were Turkish-speaking, they were translated into English for reporting, with the original meaning preserved as closely as possible. The resulting responses were analyzed using Braun and Clarke’s reflexive thematic analysis [40] with an inductive (data-driven) semantic-level orientation, following the six recursive phases: (1) familiarization with the responses; (2) generation of initial codes; (3) collation of codes into candidate themes and subthemes; (4) review of themes against the coded extracts; (5) definition and naming of themes; and (6) selection of illustrative verbatim quotations. Two researchers independently coded all responses using a shared codebook, and coding discrepancies were resolved by discussion and consensus. For each theme and subtheme, the number and proportion of participants whose responses contributed to it are reported as a transparency indicator, not as a quantitative or inferential claim. Trustworthiness was supported by independent double coding, consensus-based theme definition, retention of a coding audit trail, and reporting of verbatim quotations [40]. Given the small sample and the single-session timing of the open-ended questions, formal member checking and data saturation assessment were not undertaken; this is acknowledged among the study limitations (Section 5).

### 2.9. Kinematic Metrics: SPARC and Trajectory Efficiency

Kinematic metrics summarize how accurately, efficiently, and smoothly the hand reaches a target. They are routinely reported in post-stroke reaching studies [31,34]. Smoothness is the continuity of the velocity profile: a smooth movement has a single unfragmented velocity peak, while an impaired movement breaks into multiple sub-movements. The minimum-jerk model provides the theoretical basis for this definition [41], and SPARC has been shown to be a valid and reliable smoothness index in upper-extremity reaching [15,34,37].

**SPARC (Spectral Arc Length).** SPARC was used as the primary smoothness index. It is defined as the arc length of the amplitude-normalized velocity spectrum within a frequency cutoff ($f_{c}=10$ Hz) and an adaptive amplitude threshold (V=0.05), and approaches 0 in smooth movements while becoming more negative as movement becomes more segmented [37]. Raw SPARC values (negative scale) are reported alongside the linear 0–100 rescaling recorded by the system (${\mathrm{SPARC}}_{0-100}=100\;\cdot \;\left({\mathrm{SPARC}}_{\mathrm{raw}}+8\right)/6.5$, clipped to [0, 100]) so that higher values indicate smoother movement. This 0–100 score is a system-specific rescaling intended only for clinician-facing display; the native-scale (raw, negative) SPARC values are the appropriate quantity for cross-study comparison with the existing kinematic literature [34,37].

**Trajectory efficiency.** Trajectory efficiency was calculated as the ratio of the start-to-target straight-line distance to the actual path length (range 0–1; values closer to 1 indicate a more efficient path). The inverse, the reach-path ratio, is the more commonly reported form in the literature [31].

**Maximum deviation.** Maximum deviation was computed as the largest perpendicular distance in meters from the actual hand trajectory to the straight start–target line, and is reported in meters. The conceptually related dimensionless “index of curvature” (this perpendicular distance divided by the start–target distance) was not used as the reported quantity in this study because the start–target distance varied across game modes; reporting the absolute perpendicular distance in meters avoids confounding mode-level differences in start–target distance with movement-curvature differences. The metric is interpreted in the same direction as in [34]: smaller values indicate a straighter path.

**Target overshoot.** Target overshoot was defined as a re-increase in distance from the target after the closest approach point, and was evaluated as an indicator reflecting dysmetria [42].

Aggregation across sessions.

All kinematic outcomes were derived from the session-level values recorded by the system, each session value being the median across the completed trials in that session (computed on goal-directed reach segments—movement onset to target acquisition or release—and weighted equally across the seven FRGT modes). For each participant and outcome, a single early-phase value and a single late-phase value were then formed as the median across the five early-phase sessions (sessions 1–5) and the five late-phase sessions (sessions 16–20), respectively; these per-participant values were entered into the within-subject (Wilcoxon signed-rank) tests. The identical aggregation was applied to all primary and secondary kinematic outcomes, so that every kinematic value reported in the Results is reproducible from the session-level data in the project’s data archive.

### 2.10. Statistical Analysis

The normality assumption was evaluated with the Shapiro–Wilk test [43]; given the small sample size and non-normal distribution of several outcomes, all within-subject comparisons used the paired Wilcoxon signed-rank test, which ranks paired differences by magnitude and tests whether the median paired difference deviates from zero [44]. The two-sided significance level was set at α=0.05.

Multiple-comparisons correction (Holm–Bonferroni).

Because four pre-specified primary outcomes (success rate, mean movement speed, pause duration, SS-QOL) were tested simultaneously, the Holm–Bonferroni step-down procedure was applied to control the family-wise error rate [45]. Concretely, the four raw *p*-values were rank-ordered from smallest ($p_{\left(1\right)}$) to largest ($p_{\left(4\right)}$) and compared against the thresholds α/4, α/3, α/2, and α/1, respectively. Both raw and adjusted *p*-values are reported. The Holm–Bonferroni correction was *not* applied to secondary kinematic outcomes (SPARC, trajectory efficiency, maximum deviation, target overshoot) or to exploratory correlations; these are reported descriptively as exploratory findings.

Effect size.

For each Wilcoxon comparison, effect size *r* was calculated as $r=\left|Z\right|/\sqrt{2n}$ and interpreted using Cohen’s conventions (small ≥ 0.10, medium ≥ 0.30, large ≥ 0.50) [46]. Because effect sizes derived from very small pilot samples are known to overestimate the true effect (the so-called “winner’s curse”), reported effect sizes are explicitly flagged as upwardly biased estimates intended only for sample-size planning of a future RCT [47]. Hodges–Lehmann median differences and 95% confidence intervals are reported alongside point estimates [48].

Statistical power.

Post hoc power computed from the observed effect size of the same sample is statistically uninformative and conceptually flawed [49]; therefore, we do not report a post hoc power calculation. Instead, the observed effect sizes are presented as planning estimates for future adequately powered RCTs (see Section 6).

Comparisons between completers and non-completers; exploratory associations.

A comparison of baseline characteristics between completers and non-completers (Mann–Whitney *U* test for continuous variables; Fisher’s exact test for categorical variables) [50,51] was pre-specified but could not be carried out because baseline data for the non-completers were incomplete. Spearman rank correlations were used to characterize exploratory associations between within-subject VR-derived kinematic change and within-subject clinical change [52]. Inter-session reliability of VR metrics was evaluated using the Intraclass Correlation Coefficient (ICC), calculated over three consecutive sessions using the ICC (2,*k*) model [53].

Confounder adjustment.

Multivariable adjustment for potential confounders (age, sex, stroke chronicity, baseline function, concurrent therapy intensity) was *not* feasible given the sample size of n=10. This is acknowledged as a substantive interpretative limitation (Section 5); therefore, the within-subject changes reported here reflect the joint effect of the VR adjunct, concurrent conventional therapy, time, and natural recovery, and as such cannot be attributed to the VR system alone.

Data Preprocessing and Signal Processing

*In plain terms.* Raw hand-tracking samples contain small jitter and brief isolated dropouts. Before kinematic metrics are computed, the position signal was smoothed with a short moving-average filter (≈55 ms window at 90 Hz sampling), and the effective sampling frequency was monitored on every session to flag any unusable recording. Mathematical details follow; the full formulae for boundary correction are provided in Appendix A for replicability.

For window size *N*, the moving average filter is defined as follows:

(5) $${\bar{x}}_{i}=\frac{1}{2k+1}\sum_{j=i-k}^{i+k}x_{j}$$

where *k* is the half-window size. At the first and last *k* samples of each recording, the window is asymmetrically truncated so that no samples outside the recording are referenced; the explicit boundary correction formulae are given in Appendix A.

The default window size was N=5, providing a smoothing window of approximately 55 ms at 90 Hz sampling. This is a standard moving-average smoothing operation routinely used for time-domain noise reduction in motion tracking data [54].

The system collects data at approximately 72–90 Hz sampling frequency, depending on the Meta Quest 3 device’s frame rate. The mean sampling frequency is

(6) $$f_{\mathrm{avg}}=\frac{\left(N-1\right)}{\Delta t_{\mathrm{total}}}\times 1000\;\left[\mathrm{Hz}\right],$$

where *N* is the total sample count and $\Delta t_{\mathrm{total}}$ is the total duration in ms. Sampling-frequency quality was classified according to the bands shown in Table 2.

## 3. Results

### 3.1. Participant Flow

**Figure 5:**
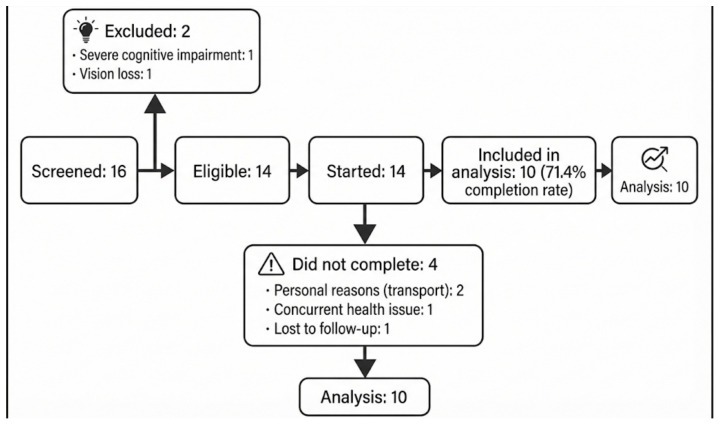
Participant flow diagram depicting screening, enrollment, intervention, and analysis phases of the 8-week single-arm pilot VR rehabilitation intervention. The study was not randomized; therefore, no allocation step is shown. Reporting follows the STROBE checklist for observational studies, with CONSORT-EHEALTH and TIDieR elements used only as guidance for the intervention description.

A total of sixteen patients were screened, of whom two did not meet the inclusion criteria and fourteen started the intervention. Of these fourteen patients, four (28.6%) did not complete the planned twenty sessions and were excluded from the per-protocol analysis; the remaining ten participants completed every one of their twenty scheduled sessions (Figure 5). Therefore, we distinguish two complementary feasibility indicators: the *intervention completion rate* (10/14 = 71.4% among those who started the intervention), and the *per-protocol session adherence among completers* (200/200 = 100% of the twenty scheduled sessions across the ten analyzed participants). The 71.4% intervention completion rate rather than the per-protocol 100% adherence figure is the principal feasibility metric, and is the value reported in the Abstract and Conclusions. Bias reduction steps included consecutive sampling, predetermined inclusion/exclusion criteria, and a standardized measurement protocol; participant flow is reported in accordance with STROBE recommendations [21]. Reasons for non-completion were logistical (transport, scheduling), low motivation, and competing time constraints; no withdrawal was attributable to a study-related adverse event. No SSQ-defined adverse events (total score >15 or any symptom rated as severe) were observed, and no participant requested early session termination due to cybersickness symptoms; the mean SSQ total score remained below 7 at all three time points (baseline, mid-protocol, and end of protocol).

Baseline characteristics of the ten completers are summarized in Table 3; a formal comparison with the four non-completers was not performed because their baseline data were incomplete. Basic participant characteristics are given in Table 4 and time since stroke distribution in Table 5.

Descriptively, the sample spanned all post-stroke phases: one participant was in the acute phase (≤1 month), two in the sub-acute phase (1–6 months), and seven in the chronic phase (>6 months, ranging up to 21 years). The within-subject changes did not follow a consistent chronicity gradient; with only one, two, and seven participants per stratum, no subgroup inference is possible. Time-dependent natural recovery is most rapid in the first weeks after stroke, and as such is expected to be largest in the acute and sub-acute strata; in this single-arm design, it is a major uncontrolled confounder that cannot be separated from any contribution of the VR adjunct or of concurrent conventional therapy. Concurrent conventional therapy details are given in Table 6; all participants continued their standard hospital-based physiotherapy program throughout the study, and the VR intervention was applied in addition to rather than in place of this treatment.

Within-subject changes between the early phase (sessions 1–5) and the late phase (sessions 16–20) are presented in Table 7. Tied zero differences were handled using the default Wilcoxon procedure (Pratt method). Holm–Bonferroni correction was applied across the four pre-specified primary outcomes; both raw and adjusted *p*-values are reported. **All four primary outcomes reached significance after Holm–Bonferroni correction ($p_{\mathrm{adj}}\leq 0.022$), but with differing levels of robustness.** Success rate, mean speed, and SS-QOL each had raw p≤0.008 and would survive even fixed Bonferroni correction across the four primary tests. **Pause duration** (raw p=0.022), by contrast, is markedly more marginal: it only reaches significance at the highest-rank Holm threshold, and would *not* survive fixed Bonferroni correction at the α/4=0.0125 level. Therefore, the pause duration result should be treated with greater caution than the other three primary outcomes. Across all four outcomes, these results are exploratory feasibility signals rather than confirmatory tests of efficacy.

Secondary (exploratory) kinematic outcomes are reported descriptively in Table 8. SPARC is reported both on its native (negative) scale as is conventional in the biomechanics literature, with values closer to 0 indicating smoother movement [34,37], and on the linear 0–100 rescaling recorded by the system. No inferential testing or multiple-comparisons correction was applied to secondary outcomes; these are exploratory, and are interpreted as descriptive trends only.

### 3.2. Standard Clinical Assessments

Standard clinical assessments were performed before and after treatment alongside VR performance data. Motor recovery was reported using Brunnstrom staging based on the upper extremity principles of the Fugl-Meyer Assessment [26,55]. Spasticity was assessed using the Modified Ashworth Scale [56]. Pain level was measured using the Visual Analog Pain Scale (VAS) [57], while quality of life was measured using the Stroke-Specific Quality of Life Scale (SS-QOL) [36].

Statistical Analysis of Clinical Scores

*Brunnstrom upper extremity stage:* Progression of at least one stage in 4/10 participants; group-level Wilcoxon Z=−2.00, raw p=0.046 (descriptive, not corrected). *Brunnstrom hand stage:* Progression in 1/10 participants; Z=−1.00, p=0.317. *Modified Ashworth Scale:* Reduction by one grade in 1/10 participants; Z=−1.00, p=0.317; no group-level change. *VAS pain:* Reduction in 2/10; Z=−1.34, p=0.180. *SS-QOL:* Increase in all ten participants; the participant-level *median* change was +18.5 points (IQR 13–42, matching Table 9), while the sample *mean* change was +27.5 points and the Hodges–Lehmann *median difference* estimate was +27.0 points (95% CI 14.0–41.0; Table 7). These three quantities address different aspects of central tendency, and should not be conflated; for the Wilcoxon test, the Hodges–Lehmann estimate is the conventional location summary. The signed-rank test yielded Z=−2.80, raw p=0.005, Holm–Bonferroni adjusted p=0.020 (Table 7). Because Brunnstrom staging is a coarse ordinal classification, these clinical results should not be interpreted as a comprehensive assessment of upper extremity motor recovery. The absence of a stroke-specific upper extremity scale with established responsiveness, such as the Fugl-Meyer Assessment Upper Extremity (FMA-UE) or the Action Research Arm Test (ARAT), is acknowledged as a substantive measurement-level limitation (see Section 5). Exploratory associations between within-subject VR-derived kinematic change and within-subject clinical change were summarized using Spearman correlations (Table 10); no inferential claim is attached to these correlations.

Exploratory Spearman correlations are presented in Table 10. Although several point estimates fell in the moderate range (ρ≈0.40–0.52), *none* reached the conventional significance threshold (p≥0.12); given the small sample (n=10), the 95% confidence intervals around these estimates are very wide and consistent with effects ranging from null to substantial. Therefore, we explicitly describe these as *non-significant trends* and *hypothesis-generating* only; no inference about the magnitude or direction of the underlying associations is intended. To avoid any over-optimistic reading: at n=10, a sample Spearman ρ≈0.50 is statistically indistinguishable from ρ=0, and the point estimates here are at least as likely to shrink toward zero in a larger sample as they are to remain in the moderate range. These correlations should be read as *candidate questions for a future RCT*, not as evidence of associations between kinematic and clinical change. Usability was good: the mean SUS score was 78.5±5.4 (range 71–86), above the 68 acceptability threshold for all ten participants and corresponding to the “good” band per Bangor and Lewis [38,58].

### 3.3. Qualitative Usability Feedback

The ten completers provided open-ended responses at the end of the protocol (Section 2.8). Reflexive thematic analysis of these responses generated three themes, each comprising two subthemes (Table 11). The themes were broadly convergent: positive responses clustered around *motivation/engagement* and the *value of feedback*, whereas critical responses clustered around concrete *design and comfort* suggestions. Theme-level participant frequencies are reported as a transparency indicator only; given that n=10, they should not be read as quantitative effect estimates.

Theme 1: Motivation and engagement (n=8, 80%).

The most frequently expressed theme was that the gamified framing made therapy enjoyable and sustainable, and comprised two subthemes. The first, *game framing reduces boredom and perceived effort*, reflected participants experiencing the sessions as play rather than as repetitive exercise: *“Doing exercises like a game was more fun; I could continue without getting bored”* (P5). The second, *scoring and self-competition as motivators*, captured the role of the in-game score in setting self-referenced goals: *“The scoring system was motivating; I wanted to beat my own score”* (P2). Together, these subthemes indicate that motivation arose both from the intrinsic enjoyment of the gamified framing and from achievement-oriented self-referenced goal setting.

Theme 2: Value of real-time and longitudinal feedback (n=7, 70%).

Participants valued being able to see their performance, both moment-to-moment and across sessions. The subtheme *immediate in-task feedback* concerned the real-time knowledge of results provided during play: *“It was nice to see immediately how well I was doing”* (P9). The subtheme *visualized progress across sessions* concerned the longitudinal progress graphs in the therapist panel: *“I could track my progress through the graphs”* (P1). This theme is consistent with motor learning principles [33], in which feedback on performance can support engagement and a sense of competence.

Theme 3: Design, content, and comfort suggestions (n=5, 50%).

Critical feedback was constructive and actionable, and formed two subthemes. The first, *desire for greater content variety*, reflected a wish for a broader range of game scenarios to sustain engagement over a multi-week program: *“There could be more game varieties”* (P3). The second, *hardware comfort and session length*, concerned the ergonomics of the head-mounted display and session duration: *“Sometimes the headset felt a bit heavy; shorter sessions would be better”* (P7). These suggestions bear directly on tolerability and on the design of the planned trial: headset weight and session length relate to physical tolerability, while content variety relates to sustaining engagement across a longer intervention. Overall, the qualitative feedback was predominantly positive and offered concrete low-cost targets for system refinement (greater content variety, attention to headset comfort, and flexible session length). The integration of these qualitative themes with the quantitative feasibility, usability, and kinematic findings is considered in Section 4.5.

## 4. Discussion and Conclusions

### 4.1. Principal Findings

This pilot study evaluated the feasibility, usability, and preliminary kinematic profile of a hand-tracking VR rehabilitation system delivered as an adjunct to conventional physiotherapy in ten completers (out of fourteen who started) with stroke. The principal feasibility findings were an intervention completion rate of 10/14 = 71.4% (per-protocol session adherence among the ten completers, 100%), no SSQ-defined adverse events, and a mean System Usability Scale score of 78.5 corresponding to the “good” usability band. Within-subject change was observed in all four pre-specified outcomes (success rate, mean speed, pause duration, SS-QOL), with Holm–Bonferroni adjusted p≤0.022. We emphasize, however, that these within-subject changes *cannot* be causally attributed to the VR system. In a single-arm design with n=10, with all participants receiving concurrent conventional physiotherapy and with three of the ten participants in the acute or sub-acute phase where natural recovery is expected, the observed changes reflect the joint effect of the VR adjunct, conventional therapy, the passage of time, and natural recovery. Therefore, the study is framed throughout as a feasibility/preliminary investigation; no claim of clinical efficacy is made or supported by the design.

### 4.2. Clinical Interpretation and Clinical Meaningfulness

Whether the within-subject change in SS-QOL is clinically meaningful cannot be established in this design: we do not claim that it meets a published responsiveness or minimal clinically-important difference threshold for the SS-QOL total score, as such a comparison would require an adequately controlled study. In any case, the observed change should not be read as evidence that the VR adjunct caused it. In our single-arm design, all participants concurrently received conventional physiotherapy; one acute and two sub-acute participants were in a phase where natural recovery alone is expected to produce SS-QOL change of similar magnitude. Therefore, it is not possible to determine what proportion of the observed change in SS-QOL is attributable to the VR adjunct, as opposed to conventional therapy, natural recovery, or expectation/Hawthorne effects. As such, the numerical comparison with the scale’s responsiveness range is descriptive only. Beyond this, two further interpretative constraints apply. First, SS-QOL is a self-report instrument; in an unblinded single-arm protocol it is susceptible to expectation, novelty, and Hawthorne effects, which cannot be disentangled from the contribution of the VR adjunct. Second, the only stroke-specific motor scale used in this study was Brunnstrom staging. Brunnstrom staging is a coarse ordinal classification, and is not designed to detect the small but clinically meaningful changes in upper-extremity function that more responsive instruments such as the Fugl-Meyer Assessment Upper Extremity (FMA-UE) and Action Research Arm Test (ARAT) are validated to detect [59,60]. The absence of these instruments is a substantive measurement-level limitation that constrains our ability to correlate continuous in-game kinematic change with discrete functional motor recovery; future controlled studies should use FMA-UE and/or ARAT alongside SS-QOL. As context only, RCTs of immersive VR added to standard care in sub-acute stroke have reported FMA-UE improvements [61], and imVR-based serious-game RCTs have shown gains in hand motor function [62]. Reported MDC_95_ values for SS-QOL physical subscales (mobility 5.9, self-care 4.0, upper extremity function 5.3) and minimal CID ranges (1.5–2.4; 1.2–1.9; 1.2–1.8, respectively) [63] indicate that in future trials reporting subscale-level change alongside the total score will strengthen clinical interpretation.

The within-subject effect sizes reported here (r=0.51–0.63) fall in the “large” band by Cohen’s convention [46], and are numerically comparable to pooled effect sizes from VR rehabilitation meta-analyses [8,64]. However, effect sizes derived from a pilot sample of n=10 are known to be upwardly biased (the “winner’s curse”), with wide confidence intervals consistent with substantially smaller true effects [47]. Therefore, we use these effect sizes only as planning estimates for sample size calculation in a future RCT, and do not interpret them as evidence of large clinical effects of the VR system per se. We do not report a post hoc power calculation, since post hoc power based on the observed effect from the same sample is statistically uninformative [49].

### 4.3. Interpretation of Kinematic Findings

The mixed SPARC findings are consistent with the well-known speed–smoothness tradeoff. As movement becomes faster, accuracy and smoothness may temporarily decrease [65]. Because SPARC indexes whether a movement is single-piece and uninterrupted [34,37], brief decreases or plateaus in SPARC during early-phase speed gains are expected, not unexpected.

Joint reporting of kinematic measures enables objective monitoring of motor control changes. In post-stroke reaching tasks, prolonged movement time, decreased peak velocity, and increased path ratio are common findings; therefore, speed, path efficiency, and smoothness are candidate process indicators for monitoring motor control, pending validation against clinical outcomes [31]. SPARC being a recommended smoothness metric strengthens the smoothness assessment in this study [34,37].

### 4.4. Usability, Safety, and Adherence

Usability findings are consistent with results from iterative design and acceptability studies published in JMIR Serious Games [9,10,11]. The SUS mean of 78.5±5.4 indicates an experience above the acceptability threshold and at a “good” usability level [38,58]. The high intervention completion (10/14 = 71.4%; 100% per-protocol session adherence among completers) and absence of cybersickness reported in this study may be evaluated as positive indicators regarding feasibility and tolerability; however, rare adverse events may have been missed due to the small sample. The literature reports negative perceptions such as difficulty in device use, dizziness/eye fatigue, and monotony in VR rehabilitation, as well as access/adherence barriers [11]. In our sample, reasons for participants who could not complete the program were reported as logistical barriers, low motivation, and time constraints; no adverse experiences (cybersickness, etc.) were reported.

### 4.5. Integration of Qualitative and Quantitative Findings

Because this was a feasibility study, the qualitative open-ended responses were collected primarily to help interpret the quantitative feasibility and usability data rather than to provide confirmation. Considering the two strands together (a convergent mixed-methods interpretation [66]) yields three points of correspondence, which are offered as explanatory context only, not as causal evidence.

First, the *motivation and engagement* theme converges with and offers a plausible experiential explanation for the quantitative adherence and usability findings. The high session-level adherence (100% of scheduled sessions among completers), absence of cybersickness-related withdrawals, and “good” SUS score (78.5) are consistent with participants’ descriptions of the system as enjoyable and as play rather than effortful exercise (Theme 1). In other words, the quantitative “what” (high adherence, good usability) and qualitative “why” (game framing, self-referenced scoring) both point in the same direction. We note, however, that 4 of 14 participants (28.6%) did not complete the program for logistical, motivational, and time-related reasons; thus, the positive engagement narrative reflects the experience of completers, and should not be generalized to all who began the intervention.

Second, the *value of feedback* theme provides a candidate mechanism for the within-subject changes observed in the in-game performance indicators (success rate and movement speed), though without establishing one. Participants explicitly valued the real-time knowledge of results during play and the visualized progress across sessions (Theme 2), which is consistent with motor-learning accounts in which augmented feedback supports practice quality and engagement [33]. We emphasize that this convergence is interpretive, as the single-arm design cannot determine whether feedback, concurrent conventional therapy, natural recovery, or expectancy effects drove the observed within-subject change.

Third, the *design, content, and comfort* theme complements the quantitative tolerability data by identifying *actionable* targets that aggregate SSQ and SUS scores cannot reveal. Although no SSQ-defined adverse event occurred, participants nonetheless flagged headset weight and a preference for shorter or more flexible sessions (Theme 3), and requested greater game variety to sustain engagement over a multi-week program. These qualitative signals are directly useful for the planned trial: they motivate attention to head-mounted-display ergonomics and session pacing (tolerability) as well as to expanding the content library (sustained engagement), and additionally illustrate the added value of collecting open-ended feedback alongside standardized instruments. Taken together, the qualitative and quantitative strands are mutually reinforcing at the level of feasibility and usability, although neither strand—either individually or jointly—can support inference about clinical efficacy in this uncontrolled design.

### 4.6. Comparison with the Literature and Technical Considerations

Existing upper-extremity VR rehabilitation systems generally use controller-based interaction or external sensors (e.g., Leap Motion) [12,13,14]. Meta Quest 3’s integrated hand-tracking technology eliminates the need for controllers, thereby increasing accessibility for stroke patients experiencing grip difficulties. Technical findings indicating that hand-tracking accuracy is sufficient for kinematic analysis support the applicability of our measurements [16,17,18]. This comparison is summarized in Table 12.

Bressler and colleagues’ iterative design study [10] similarly used VR for hand and finger rehabilitation, but worked with a different hand-tracking technology. The SUS score of 78.5 observed in our study is consistent with the “good” category in that study. Campos and colleagues [16] found the Meta Quest 3’s hand-tracking accuracy to be suitable for upper-limb kinematic analysis, supporting the technical validity of our measurements. Systematic reviews and clinical studies in Leap Motion-based upper extremity rehabilitation report functional gains and trends pointing toward high engagement/satisfaction [12,13,14].

### 4.7. Baseline Performance and Change Relationship

The descriptive pattern in this small pilot—larger within-subject change in participants with lower baseline in-game performance (ρ≈−0.71) and in participants with a shorter time since stroke—is consistent with two non-exclusive explanations that this design cannot separate: *(i)* a genuine ceiling-effect/headroom-for-improvement effect in which participants who started further from the maximum in-game score have, by definition, more room to improve, irrespective of any specific contribution of the VR adjunct; and *(ii)* a natural-recovery effect in which spontaneous post-stroke motor recovery is itself largest in the first weeks to months and in patients with greater initial impairment, so the same baseline–change association is expected even without any intervention. Therefore, we do *not* interpret the ρ≈−0.71 as evidence that the VR adjunct is differentially effective in lower-baseline or earlier-stage patients. It is a descriptive observation, fully consistent with regression-to-the-mean and natural recovery. Any inference about differential effectiveness would require a randomized trial with stroke chronicity and baseline FMA-UE/ARAT as pre-specified stratification factors. Similarly, the observation of SS-QOL increases in the older participant (H7, 77 years) and in the two participants with the longest time since stroke (H8, 9 years; H10, 21 years) is reported only descriptively and as hypothesis-generating; in an n=10 sample with no control group, single-participant observations cannot support inference about effectiveness in elderly or chronic patients.

The wider context for the within-subject heterogeneity of this pilot is that the sample combined three sources of variability that future trials should consider stratifying on: *(a)* stroke chronicity (acute ≤1 month, n=1; sub-acute 1–6 months, n=2; chronic >6 months, n=7, ranging up to 21 years); *(b)* baseline upper-extremity motor impairment (Brunnstrom upper stage 3–5c, with hand stage 3–5); and *(c)* age (27–77 years). With only ten participants, these three sources cannot be analytically disentangled. As a consequence, the kinematic “trends” presented in this manuscript reflect not only any contribution of the VR adjunct but also the mixed distribution of these patient-level factors. Future trials with adequate sample size should stratify randomization on stroke chronicity and baseline motor impairment so that within-stratum effects of the VR adjunct can be estimated separately.

### 4.8. Conclusions and Future Work

Overall, this pilot study supports the *feasibility* of delivering a controller-free hand-tracking VR rehabilitation system as an adjunct to conventional physiotherapy in individuals with stroke: an intervention completion rate of 10/14 = 71.4% (100% per-protocol session adherence among completers), no SSQ-defined adverse events, and “good” SUS-rated usability indicate that the protocol is implementable in routine inpatient rehabilitation. We do not interpret the observed within-subject changes as evidence of clinical efficacy. Because the design is single-arm, the sample is small (n=10), and all participants concurrently received conventional therapy, the observed changes cannot be causally attributed to the VR system and should be regarded as exploratory feasibility signals rather than treatment effects. Validation against gold-standard motion capture, characterization of measurement error in the SPARC pipeline, and sensitivity analyses are also recommended before in-game kinematic indicators are used for clinical decision-making [17,18]. In summary, the hand-tracking VR rehabilitation system was feasible, well tolerated, and rated as having good usability when delivered as an adjunct to standard care. Adequately powered randomized controlled trials with stroke-specific motor scales (FMA-UE, ARAT) and analytical control of conventional therapy intensity are required before any conclusion regarding clinical effectiveness can be drawn.

## 5. Limitations

This study has several substantive limitations that constrain the interpretation of its findings. Consistent with the feasibility framing of the study, several of these reflect deliberate features of a single-arm pilot design rather than remediable flaws; for clarity, and in response to the editorial request to review the limitations for relevance, they are grouped thematically into five clusters rather than presented as an undifferentiated list. The most consequential cluster for interpretation is the first (design and confounding), which is why no causal claim is made anywhere in the manuscript.

1. **Design and confounding (no causal inference possible).** The single-arm pre–post design provides no counterfactual against which to evaluate the VR adjunct, and all ten participants concurrently received standard hospital-based physiotherapy (median three sessions/week; ∼45 min/session; Table 6). Consequently, whether the observed within-subject changes reflect any contribution of the VR system, the concurrent conventional physiotherapy, time-dependent natural recovery (especially in the one acute and two sub-acute participants), Hawthorne effect, or expectancy/novelty effects *cannot be determined*, and the changes must not be interpreted as treatment effects of the VR system. Intensity of conventional therapy was neither standardized nor protocolized (content: ROM, strengthening, neurophysiological exercise) and could not be entered as a covariate, and multivariable adjustment for age, sex, stroke chronicity, baseline function, and concurrent-therapy intensity was not feasible at n=10. Neither participants nor therapists could be blinded. Therefore, we avoid any phrasing (e.g., “VR was associated with improvement”) that could imply causality, and report only within-subject change. A two-arm, assessor-blinded randomized controlled trial with analytic control of conventional therapy intensity is the appropriate next step [19,20].
2. **Sample size, heterogeneity, and generalizability.** The sample (ten completers of the fourteen who started) provides limited precision: the 95% confidence intervals around the reported effect sizes are wide and consistent with effects ranging from null to substantial, and effect sizes from very small pilot samples are systematically inflated (the “winner’s curse”) [47]. Therefore, the reported r=0.51–0.63 should be used *only* as planning input for a future RCT, not as evidence of a large clinical effect, and—following [49]—no post hoc power computed from the observed effect is reported. The wide ranges in age (27–77 years) and time since stroke (1 month–21 years), although representative of routine case mix, preclude meaningful subgroup analysis at n=10. In addition, the single-center setting limits external validity and does not capture inter-center variability in conventional therapy and case mix.
3. **Measurement and outcome limitations.** The only motor recovery instruments were Brunnstrom upper-extremity and hand staging, which are coarse, categorical, and lack the responsiveness of the FMA-UE or ARAT [59,60]. This constrains the correlation of continuous in-game kinematic change with sensitive, validated functional motor outcomes; future controlled studies will adopt FMA-UE and ARAT as primary motor outcomes alongside SS-QOL. In addition, consumer-grade Quest 3 hand tracking was not validated in-protocol against a gold-standard optical motion capture system, meaning that the reported kinematic values inherit any device-level bias despite the centimeter-level accuracy characterized in prior bench studies [16,17,18]. Cybersickness was monitored with the validated Simulator Sickness Questionnaire [39] at three predefined time points and on spontaneous report, but not after every session; although no SSQ-defined adverse events occurred, rare events may have been missed at n=10.
4. **Procedural and analytic limitations.** As a pilot feasibility investigation, the study was not prospectively registered; future confirmatory studies will be prospectively registered (e.g., on ClinicalTrials.gov) before recruitment begins. Formal sensitivity analyses (e.g., to alternative pause detection thresholds, smoothing window sizes, and exclusion of advanced modes) were beyond the scope of this pilot and will be pre-specified in the analysis plan of the planned RCT.
5. **Qualitative component.** The qualitative strand was deliberately brief: it was based on two open-ended questions administered at a single time point (end of protocol) to the ten completers, and was intended to contextualize rather than independently establish the usability findings. Formal member checking and data-saturation assessment were not undertaken, and the perspectives of non-completers were not captured. Richer qualitative methods (e.g., semi-structured interviews conducted across the intervention period) are planned for the future trial.

## 6. Hypotheses for Future Investigation and RCT Design Considerations

This section is intentionally framed as a set of *hypotheses to be tested in future controlled studies*, not as recommendations for current clinical practice. An uncontrolled, single-arm, per-protocol feasibility study with n=10 completers cannot support clinical practice recommendations regarding patient selection, intervention timing, or choice of outcome metric; those questions can only be answered in adequately powered randomized controlled trials. Therefore, the bullets below are presented as *candidate design parameters and hypotheses* for the planned RCT.

1. **Candidate eligibility profile to be tested:**
  - Inclusion (hypothesis): Brunnstrom upper-extremity stage 3–5, active finger extension present, MMSE ≥ 24/30.
  - Subgroup to examine a priori (hypothesis): Patients with lower baseline in-game performance, who in this pilot showed a within-subject inverse association between baseline performance and within-subject change. This is not a clinical practice recommendation; the underlying association (r≈−0.71) is itself derived from n=10, and may regress to the null in a larger sample.
  - Pre-specified safety screening for severe spasticity (Ashworth ≥3), severe visual or hearing impairment, vestibular problems, uncontrolled epilepsy, active psychosis, or severe motion sickness history, to be assessed during eligibility screening for the future RCT.
2. **Intervention-timing hypotheses (to be tested, not recommended for practice):**
  - This pilot included only one acute and two sub-acute participants, and showed no consistent chronicity gradient; thus, it cannot indicate whether response differs by post-stroke phase. Both the early neuroplasticity window *and* natural recovery would predict phase-dependent differences, and the two cannot be separated in a single-arm design.
  - Future stratified-randomization or pre-specified subgroup analyses in an RCT, with stroke chronicity as a stratification factor, would be required before any claim about preferential effectiveness in early phases can be made.
3. **Candidate process measures to be evaluated in an RCT:** Movement speed and pause duration showed the most consistent within-subject change in this pilot and are therefore candidate process measures to be evaluated against gold-standard clinical scales in the future RCT. We do *not* recommend their adoption in routine clinical assessment on the basis of this pilot.
4. **Session intensity parameters used in this pilot (for replication):** 2–3 sessions per week, 15–20 min per session, 20 sessions over ∼8 weeks. These parameters were tolerable in this pilot (intervention completion 10/14 = 71.4%; no SSQ-defined adverse events; mean SSQ total <7) and are reported here only to enable replication. They should not be inferred to be the optimal dose. Meta-analyses suggest that approximately 16 h of additional therapy in the first 6 months are associated with small but significant effects on ADL and walking [67]; the optimal VR-specific dose remains an open question.

The consumer-grade pricing and portability of the Meta Quest 3 increase the practical feasibility of larger VR rehabilitation trials in both clinical and home-based settings; the lower hardware cost compared with traditional robotic rehabilitation may make adequately powered RCTs achievable in resource-limited healthcare systems. Whether the controller-free hand-tracking modality *actually* confers a clinically meaningful advantage for patients with grip impairment remains a hypothesis to be tested.

Based on this pilot, the following research directions are planned:

1. **Randomized Controlled Trial (RCT) Design:** In the next phase, a parallel-group, single-blind (assessor-blinded), randomized controlled trial is planned.
  - Design: Parallel-group, single-blind (assessor blinding), stratified by stroke chronicity (acute/sub-acute/chronic).
  - Sample size calculation (basis): The future-RCT sample size will *not* be calculated from the observed pilot effect size (r≈0.60) directly, because effect sizes derived from n=10 are systematically inflated [47,49]. Instead, sample size will be anchored to the minimum clinically important difference (MCID) for the FMA-UE (approximately 5.25 points; standard deviation ≈10 points in published stroke samples [59]) or, as a sensitivity analysis, to a conservatively shrunken pilot effect (e.g., r≈0.30, a medium effect, after de-biasing the observed r≈0.60).
  - Sample size calculation (parameters): Two-sided α=0.05, statistical power 1−β=0.80 (i.e., type II error β=0.20), 15–20% expected dropout. Under an FMA-UE-anchored MCID-based calculation, this yields approximately N≈ 80–120 participants in total (40–60 per arm; G*Power 3.1 [68]). The previously reported figure of N≈50–60 was derived from the inflated pilot effect size of r≈0.60, and is consequently retracted from this revised plan.
  - Primary outcome measures: Fugl-Meyer Assessment Upper Extremity (FMA-UE) [59] and Action Research Arm Test (ARAT) [60,69] alongside the pilot in-game kinematic indicators.
  - Secondary outcome measures: SPARC, trajectory efficiency, SS-QOL, SUS.
  - Randomization: Computer-based block randomization (block size = 4–6).
  - Blinding: Assessor blinding (independent assessor for clinical outcomes).
  - Groups: Standard rehabilitation (A) and standard rehabilitation + VR adjunct (B).
  - Follow-up: At 3 and 6 months post-intervention.
  - Registration: Prospective registration at ClinicalTrials.gov before enrollment of the first participant.
2. **Long-term Follow-up:** Long-term persistence of VR treatment effects will be examined with 3, 6, and 12-month follow-up assessments. This is critical, especially for motor learning consolidation and transfer to functional activities.
3. **Home-Based Rehabilitation:** The portability of Meta Quest devices enables the development of home-based and self-directed rehabilitation programs [70]. The potential clinical benefit of remote treatment protocols under therapist supervision with a tele-rehabilitation model will be investigated. Current reviews report promising results of VR and AR technologies in post-stroke rehabilitation [71]. Recent umbrella reviews and eHealth-based systematic reviews evaluating the effect of tele-rehabilitation on motor function and quality of life report clinical benefit and acceptability under appropriate conditions [72,73].
4. **Personalized Adaptation:** Dynamic difficulty adjustment based on patient performance and individualized treatment protocols using machine learning algorithms are planned.
5. **Neuroimaging:** Examination of cortical reorganization markers with functional MRI will contribute to understanding the effects of VR rehabilitation on neuroplasticity [74].

In summary, this pilot feasibility study indicates that the hand-tracking VR rehabilitation system, delivered as an adjunct to conventional physiotherapy, was feasible and well tolerated in individuals with stroke (SUS =78.5±5.4; intervention completion rate 10/14 = 71.4%; per-protocol session adherence among completers 100%; no SSQ-defined adverse events). Within-subject changes between early and late phase were observed for success rate (+10.6 points, raw p=0.005, Holm-adjusted p=0.020), mean movement speed (+0.10 m/s, raw p=0.008, adjusted p=0.020), pause duration (−341 ms, raw p=0.022, adjusted p=0.022), and SS-QOL (Hodges–Lehmann median difference +27.0 points; participant-level median Δ=+18.5; raw p=0.005, adjusted p=0.020). Because this study used a single-arm design, included only ten participants, and did not control the effect of concurrent conventional therapy, these within-subject changes *cannot* be causally attributed to the VR system; they are exploratory feasibility signals only. The findings support the planning, but not the conclusion, of a future RCT in which the marginal contribution of the VR adjunct over standard care can be tested with FMA-UE and/or ARAT as primary clinical outcomes alongside in-game kinematic indicators. Within the broader literature, both non-immersive VR multi-center RCTs [75] and controlled upper-extremity VR studies [76] report variable functional gains; future analyses should distinguish “recovery” from “compensation” [77] and draw on reviews of immersive VR in healthcare [78], exergame usability [79], corticospinal plasticity [80], and post-injury recovery mechanisms [81] to interpret potential effect pathways.

## Figures and Tables

**Table 1:** FRGT (Functional Reach & Grasp Task) modes (1–4: basic game modes; 5–7: advanced game modes).

#	Mode	Screenshot	Description
1	Reach	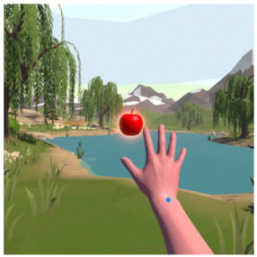	**Objective:** Reaching the target through simple reaching motion. **Mechanics:** Fixed or sequential targets appear; task is completed when hand touches the target. **Adjustable Parameters:** Number of targets, target arrangement, time limit, success criterion (touch).
2	Grip	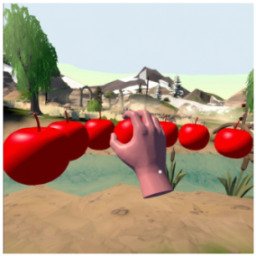	**Objective:** Activating finger motor control and grip strength. **Mechanics:** Apple/object is grasped and held; successful grip requires holding for a specified duration. **Adjustable Parameters:** Hold duration, object size, grip strength threshold.
3	Carry	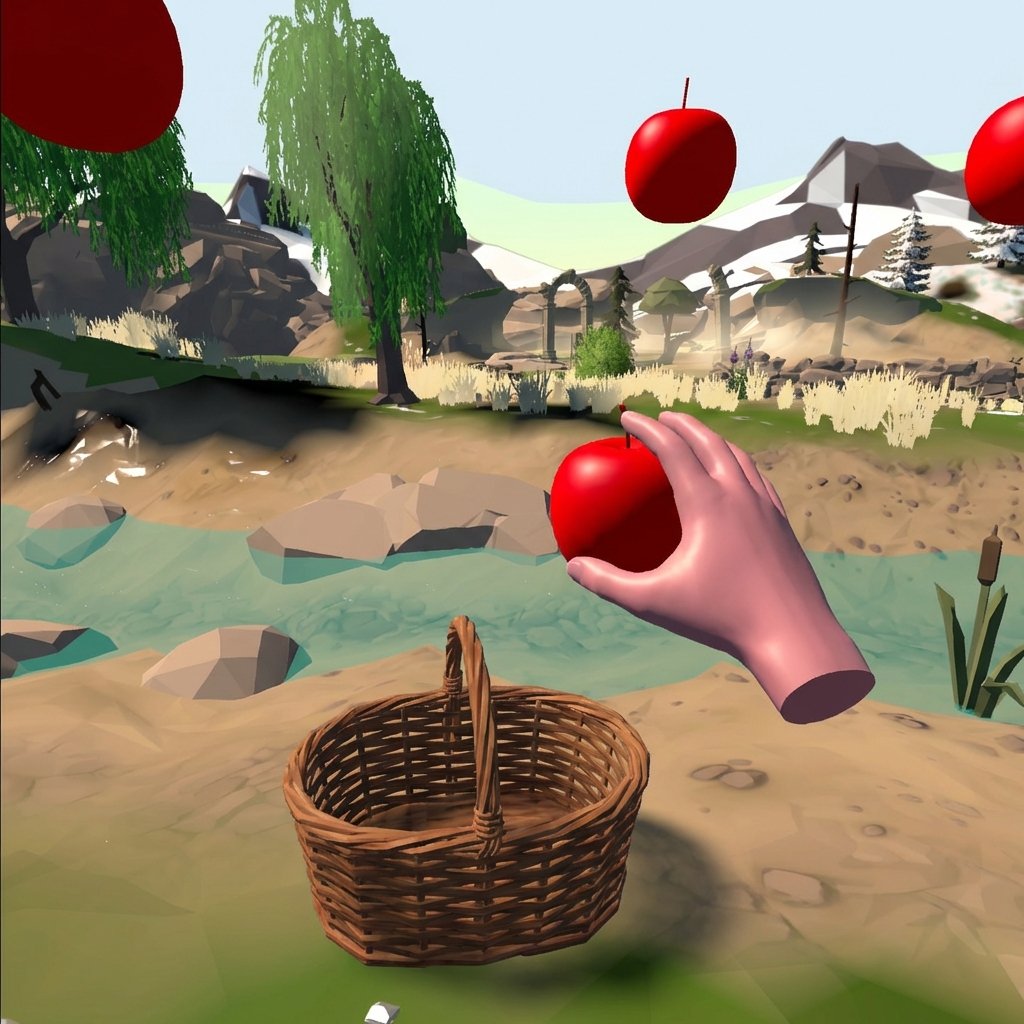	**Objective:** Coordination of carrying and releasing after catching. **Mechanics:** Object is caught, carried to the basket, and released; all stages must be completed. **Adjustable Parameters:** Basket distance, carry path, drop zone size.
4	Sort	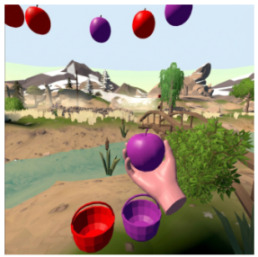	**Objective:** Cognitive-motor integration through color/shape discrimination. **Mechanics:** Objects of different colors are placed in the correct basket; incorrect placement results in point loss. **Adjustable Parameters:** Number of colors, object count, basket arrangement.
5	Reflex	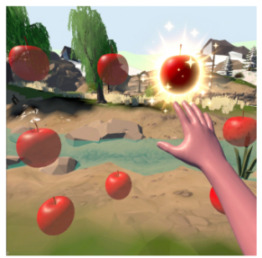	**Objective:** Reaction time and rapid motor response. **Mechanics:** Quick touch/grasp to briefly appearing target; delayed response counts as failure. **Adjustable Parameters:** Target display duration, inter-target interval, response window.
6	Moving Targets	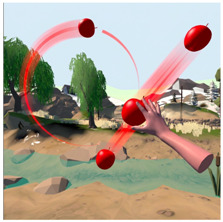	**Objective:** Dynamic target tracking and timing. **Mechanics:** Targets move; user attempts to catch the target. **Adjustable Parameters:** Movement speed, trajectory pattern, target size.
7	Glass Cube	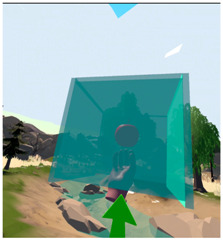	**Objective:** Precise hand positioning and collision control. **Mechanics:** Object is carefully retrieved from inside the glass cube; hitting the cube is recorded as an error. **Adjustable Parameters:** Cube size, object position, collision sensitivity.

**Table 2:** Sampling frequency quality classification and clinical suitability.

Quality	Frequency Range	Clinical Suitability
Excellent	$f_{\mathrm{avg}}>60$ Hz	Suitable for all analyses
Good	$30<f_{\mathrm{avg}}\leq 60$ Hz	Suitable for most analyses
Moderate	$15<f_{\mathrm{avg}}\leq 30$ Hz	Basic metrics can be calculated
Low	$f_{\mathrm{avg}}\leq 15$ Hz	Reliable analysis not possible

**Table 3:** Baseline characteristics of the ten completers.

Variable	Value
Age (years), median (IQR)	53.0 (46–59)
Sex (male)	70% (7/10)
Time since stroke (months), median (IQR)	19.5 (2–96)
Brunnstrom upper stage, median	4
Baseline SS-QOL, median (IQR)	131 (108–184)

Baseline data for the four participants who did not complete the intervention were incomplete, so a formal comparison of completers vs. non-completers was not performed; non-completion was attributable to logistical, motivational, and time-related factors rather than to any study-related adverse events.

**Table 4:** Participant characteristics.

Variable	Value
Age (years), mean ± SD	51.6±14.1
Age range (years)	27–77
Sex	7 Male (70%), 3 Female (30%)
Stroke type	7 Ischemic (70%), 3 Hemorrhagic (30%)
Affected side	6 Right (60%), 4 Left (40%)

**Table 5:** Time since stroke distribution.

Category	Definition	*n* (%)	Median (Range)
Acute	≤1 month	1 (10%)	1.0 month
Subacute	1–6 months	2 (20%)	1.75 (1.5–2.0) months
Chronic	>6 months	7 (70%)	30 (12–252) months
Total	—	10 (100%)	19.5 (1–252) months

**Table 6:** Concurrent conventional therapy details.

Parameter	Value
Weekly session count, median (IQR)	3 (2–5)
Session duration (minutes), mean ± SD	45±10
Treatment content	Neurophysiological exercise, ROM, strengthening
Change in treatment intensity	Stable (80%), variable (20%)
VR session scheduling	Separate day/time from conventional therapy

**Table 7:** Within-subject change from early phase (sessions 1–5) to late phase (sessions 16–20) for the four pre-specified primary outcomes. Adjusted *p*-values use Holm–Bonferroni correction across the four primary tests.

Measure	Early (Median, IQR)	Late (Median, IQR)	Median Diff ^b^ (95% CI)	*Z*	$p_{\mathrm{raw}}$ ^a^	$p_{\mathrm{adj}}$ ^d^	*r* ^c^
Success rate (%)	85.8 (80.7–89.6)	95.8 (93.3–99.2)	+10.6 (7.6–13.1)	−2.80	0.005	0.020	0.63
Mean speed (m/s)	0.29 (0.21–0.33)	0.37 (0.29–0.51)	+0.10 (0.04–0.18)	−2.67	0.008	0.020	0.60
Pause duration (ms)	558 (178–1327)	307 (158–692)	−341 (−688 to −73)	−2.29	0.022	0.022	0.51
SS-QOL	131.0 (108–184)	153.0 (140–218)	+27.0 (14.0–41.0)	−2.80	0.005	0.020	0.63

^a^ Raw two-sided Wilcoxon signed-rank test *p*-value (paired). ^b^ Hodges–Lehmann median difference estimate with 95% confidence interval [48]. ^c^ Effect size $r=\left|Z\right|/\sqrt{2n}$, n=10. Effect sizes derived from n=10 are upwardly biased and should be treated as planning estimates only [47,49]. ^d^ Holm–Bonferroni adjusted *p*-value across k=4 pre-specified primary comparisons (success rate, mean speed, pause duration, SS-QOL). Adjusted thresholds are α/4,α/3,α/2,α/1 in step-down order.

**Table 8:** Secondary (exploratory) kinematic outcomes. Raw values are reported on the native scales conventional in the biomechanics literature; transformed values are reported on a clinician-facing scale.

Measure	Early Phase	Late Phase	Change
SPARC, raw (unitless; closer to 0 = smoother)	−3.07	−3.05	+0.02
SPARC, transformed (0–100; higher = smoother)	75.9	76.1	+0.2
Trajectory efficiency (%; higher = more efficient)	57.3	62.4	+5.1
Target overshoot (count/session; lower = better)	0.25	0.20	−0.05
Maximum deviation (m; lower = straighter path)	0.114	0.110	−0.004 (−4%)

**Table 9:** Standard clinical assessment results (pre → post) at the participant level. Δ columns and group-level Wilcoxon signed-rank results are shown for each clinical scale; secondary clinical comparisons are reported descriptively without multiple-comparisons correction.

Patient	VAS (Δ)	Br. Upper (Δ)	Br. Hand (Δ)	Ashworth (Δ)	SS-QOL (Δ)
H1	0 → 0 (0)	4b → 5b (+1)	4 → 5 (+1)	1 → 0 (−1)	207 → 219 (+12)
H2	0 → 0 (0)	3 → 4a (+1)	3 → 3 (0)	2 → 2 (0)	186 → 218 (+32)
H3	0 → 0 (0)	3 → 4a (+1)	3 → 3 (0)	2 → 2 (0)	134 → 148 (+14)
H4	3 → 0 (−3)	3 → 3 (0)	3 → 3 (0)	2 → 2 (0)	108 → 121 (+13)
H5	0 → 0 (0)	5c → 5c (0)	4 → 4 (0)	0 → 0 (0)	84 → 153 (+69)
H6	0 → 0 (0)	3 → 3 (0)	3 → 3 (0)	2 → 2 (0)	139 → 153 (+14)
H7	0 → 0 (0)	4c → 5b (+1)	4 → 4 (0)	0 → 0 (0)	104 → 127 (+23)
H8	0 → 0 (0)	5c → 5c (0)	5 → 5 (0)	1 → 1 (0)	184 → 228 (+44)
H9	5 → 0 (−5)	5c → 5c (0)	5 → 5 (0)	0 → 0 (0)	119 → 161 (+42)
H10	0 → 0 (0)	5c → 5c (0)	5 → 5 (0)	0 → 0 (0)	128 → 140 (+12)
**Median Δ (IQR)**	0 (0–0)	0 (0–+1)	0 (0–0)	0 (0–0)	+18.5 (13–42)
**Wilcoxon Z**	−1.34	−2.00	−1.00	−1.00	−2.80
**Raw p**	0.180	0.046	0.317	0.317	0.005
**Imp./unch./wors.**	2/8/0	4/6/0	1/9/0	1/9/0	10/0/0

VAS: Visual Analog Pain Scale; Br. Upper: Brunnstrom Upper Extremity; Br. Hand: Brunnstrom Hand; Ashworth: Modified Ashworth Scale; SS-QOL: Stroke-Specific Quality of Life Scale. SS-QOL Wilcoxon *p* remains the primary inferential analysis (Table 7); other clinical comparisons are reported descriptively. Where the source assessment recorded a Brunnstrom range (e.g., “5–6”), a single representative sub-stage was used for tabulation; this affects only the per-participant display and not the group-level result.

**Table 10:** Exploratory Spearman rank correlations between within-subject VR-derived kinematic change and within-subject clinical change. No multiple-comparisons correction was applied to these exploratory correlations (consistent with the pre-specified analysis plan in Section 2.10, which limited Holm–Bonferroni correction to the four pre-specified primary outcomes). None of the correlations reached the unadjusted two-sided α=0.05 threshold (smallest raw p=0.12); therefore, all values are reported as exploratory hypothesis-generating and non-significant trends only.

VR Metric (Δ)	SS-QOL (Δ)	Brunnstrom Upper (Δ)	Brunnstrom Hand (Δ)
Mean speed	ρ=0.42 (p=0.22)	ρ=0.38 (p=0.28)	ρ=0.21 (p=0.56)
Success rate	ρ=0.51 (p=0.13)	ρ=0.44 (p=0.20)	ρ=0.18 (p=0.62)
Trajectory eff.	ρ=0.47 (p=0.17)	ρ=0.52 (p=0.12)	ρ=0.29 (p=0.42)
SPARC	ρ=0.31 (p=0.38)	ρ=0.28 (p=0.43)	ρ=0.15 (p=0.68)

**Table 11:** Reflexive thematic analysis of end-of-protocol open-ended responses (n=10 completers): themes, subthemes, and illustrative verbatim quotations. Participant frequencies (*n*, %) are reported as a transparency indicator only, and are not quantitative or inferential estimates.

Theme (*n*, %)	Subtheme	Illustrative Verbatim Quotation
**1. Motivation and engagement** (n=8, 80%)	1a. Game framing reduces boredom and perceived effort	“Doing exercises like a game was more fun; I could continue without getting bored.” (P5)
	1b. Scoring and self-competition as motivators	“The scoring system was motivating; I wanted to beat my own score.” (P2)
**2. Value of feedback** (n=7, 70%)	2a. Immediate in-task feedback	“It was nice to see immediately how well I was doing.” (P9)
	2b. Visualized progress across sessions	“I could track my progress through the graphs.” (P1)
**3. Design, content, and comfort suggestions** (n=5, 50%)	3a. Desire for greater content variety	“There could be more game varieties.” (P3)
	3b. Hardware comfort and session length	“Sometimes the headset felt a bit heavy; shorter sessions would be better.” (P7)

Quotations are reproduced verbatim from participant responses (translated from Turkish; original meaning preserved). P1–P10 are anonymized qualitative-respondent identifiers.

**Table 12:** Comparison of Meta Quest 3 with controller-based VR and Leap Motion.

Feature	This Study (Meta Quest 3)	Controller-Based VR	Leap Motion Based
Hardware cost	Medium (consumer-grade)	Medium–High	Low–Moderate
Setup complexity	Low (single device)	Low	Moderate (calibration)
Finger-level tracking	Yes (26 points/hand)	No	Yes
Wireless operation	Yes	Yes	Limited
Motor-impaired user compatibility	High (controller-free)	Low (grip required)	High

## Data Availability

Individual-level clinical and kinematic data generated in this study are not publicly shared due to participant privacy and institutional policies. Anonymized summary data and analysis scripts are available from the corresponding author upon reasonable request.

## Abbreviations

The following abbreviations are used in this manuscript:

ADL	Activities of Daily Living
API	Application Programming Interface
ARAT	Action Research Arm Test
CI	Confidence Interval
CID	Clinically Important Difference
CONSORT-EHEALTH	CONSORT Extension for Digital Health Interventions
FMA-UE	Fugl-Meyer Assessment—Upper Extremity
FRGT	Functional Reach & Grasp Task
HMD	Head-Mounted Display
ICC	Intraclass Correlation Coefficient
IQR	Interquartile Range
RCT	Randomized Controlled Trial
ROM	Range of Motion
SD	Standard Deviation
SPARC	Spectral Arc Length
SS-QOL	Stroke-Specific Quality of Life
SUS	System Usability Scale
VAS	Visual Analog Pain Scale
VR	Virtual Reality

## Appendix A. Technical Supplementary Details

This appendix contains derivations and boundary-handling formulae that were moved out of the main text to improve readability for clinical readers while retaining full mathematical detail for replication.

### Appendix A.1. Moving-Average Filter with Boundary Correction

For a centered moving average of half-window *k* applied to a length-*N* recording, the boundary-corrected estimate at index *i* is

(A1) $${\bar{x}}_{i}=\frac{1}{j_{\mathrm{end}}-j_{\mathrm{start}}+1}\sum_{j=j_{\mathrm{start}}}^{j_{\mathrm{end}}}x_{j},$$

(A2) $$j_{\mathrm{start}}=\max\left(0,\;i-k\right),\;j_{\mathrm{end}}=\min\left(N-1,\;i+k\right).$$

At the first and last *k* samples, the window is asymmetrically truncated so that no out-of-recording samples are referenced; in the interior of the recording, the filter reduces to the standard symmetric form given as Equation (5) in the main text.

### Appendix A.2. Effective Sampling Frequency Check

The system continuously monitors the effective sampling frequency. For a recording containing *N* samples spanning a total duration of $\Delta t_{\mathrm{total}}$ ms, the mean effective sampling frequency is

(A3) $$f_{\mathrm{avg}}=\frac{\left(N-1\right)}{\Delta t_{\mathrm{total}}}\times 1000\;\left[\mathrm{Hz}\right].$$

This value is computed per session and classified according to the bands in Table 2 (main text). Recordings classified as “Low” ($f_{\mathrm{avg}}\leq 15$ Hz) would have been excluded from the analysis; however, no recording met that criterion in this study.

### Appendix A.3. Reference Frame Transformation

Equations (2)–(4) in the main text define the rotation, homogeneous transformation, and world-to-reference operations used to express raw hand-tracking samples in the patient-centered reference frame. These are standard rigid-body kinematics operations [30], and are reproduced in the main text rather than here so that the patient-centered coordinate assumption is visible to the reader in the Methods section. No additional derivation is required beyond what is shown in the main text.
